# How, When and Why Is Job Insecurity Associated with Customer Orientation of Employees? A Mixed-Methods Investigation in the Tourism Industry

**DOI:** 10.3390/bs16101861

**Published:** 2026-10-09

**Authors:** Juan Liu, Jun Liu, Wei Wei

**Affiliations:** 1 School of Digital Economics and Management, Wuxi University, Wuxi 214105, China; liuj@wxu.edu.cn; 2 Rosen College of Hospitality Management, University of Central Florida, Orlando, FL 32819, USA

**Keywords:** job insecurity, customer orientation, work engagement, gender, industry tenure

## Abstract

The rapid advancement of technologies and the lasting impact of the COVID-19 pandemic have exacerbated the job insecurity of tourism employees. This study aims to investigate how, when and why job insecurity is associated with customer orientation of tourism employees with different genders and industry tenures. A mixed-methods approach, encompassing a quantitative survey succeeded by a qualitative inquiry, was adopted to acquire an in-depth understanding of the study relationships. The quantitative results reveal that job insecurity is negatively and indirectly associated with customer orientation through decreased work engagement; job insecurity is also directly associated with customer orientation, but the direct path is only significant among female employees whose industry tenure is at or below the sample mean. The qualitative study then explored plausible reasons behind the quantitative results. The results enrich the understanding of the association between tourism employees’ job insecurity and its outcomes, and provide a theoretical foundation and practical guidance for differentiated management strategies for motivating tourism employees under the current prevailing atmosphere of job insecurity.

## 1. Introduction

The rapid advancement of artificial intelligence (AI) technologies has not only improved the service efficiency of the tourism industry, but also has increased the possibility of the tourism workforce being replaced by AI (Tan et al., 2024). The COVID-19 pandemic has brought tremendous pressure and challenges to the economic development and employment situation worldwide, and such challenges faced by the tourism industry have been further exacerbated (Colmekcioglu et al., 2022). The intertwining of AI advancement, the pandemic and the economic turndown has instilled an atmosphere of job insecurity among tourism employees (Parvez et al., 2022).

Job insecurity refers to the feeling of fear and threat experienced by employees when facing potential job loss (Ashford et al., 1989). Given that a significant number of roles in the tourism industry are characterized by low skill requirements and are susceptible to automation through rapidly advancing AI, job insecurity among tourism employees may be more widespread and intense. The psychological and behavioral consequences of job insecurity have provoked debate. One stream of research rooted in the Stress Appraisal Theory (Lazarus & Folkman, 1984) posits that job insecurity is perceived by employees as a hindrance stressor (Currie et al., 2023; Tuan, 2022) and thereby brings detrimental impacts on employee psychology and behavior, such as higher emotional exhaustion (Dogantekin et al., 2022), poorer mental health (Gupta & Dhar, 2024), decreased affective commitment (Haldorai et al., 2024), inferior service performance (He et al., 2023) and more withdrawal behaviors (Khan et al., 2022). Another stream of studies enlightened by the Conservation of Resource Theory (Hobfoll, 2011) and impression management perspective (Shoss et al., 2023) believes that with potential job loss (resource), employees strive to invest more resources (e.g., time and effort) to preserve their current resources (job), that is, they are pushed by job insecurity to work harder (Probst et al., 2007) and perform better (Hewlin et al., 2016). Given the inconsistent findings and the prevalent sense of job insecurity within the tourism industry, there is a substantial need for research that delves into the psychological and/or behavioral repercussions of job insecurity (Haldorai et al., 2024).

Despite this debate, prior research has largely examined either the direct or the indirect effect of job insecurity, and has rarely modeled both simultaneously or specified the boundary conditions under which the direct association remains. This leaves the question of why impairing and motivating accounts can both receive empirical support unresolved (Lazarus & Folkman, 1984; Hobfoll, 2011; Shoss et al., 2023). The present study addresses this question by distinguishing an indirect pathway through work engagement from a direct pathway that reflects reduced discretionary customer-oriented effort, and by examining whether gender and industry tenure jointly condition the direct pathway. A mixed-methods design is used to explain the mechanisms underlying the quantitative pattern.

This study makes three theoretical contributions. First, it reconciles inconsistent findings by modeling direct and indirect pathways simultaneously, showing that job insecurity can impair customer orientation through reduced work engagement while also exerting a conditional direct effect. Second, it identifies gender and industry tenure as joint boundary conditions, specifying which employees are most vulnerable to the direct effect. Third, it uses qualitative accounts to explain the perceived-return mechanism and the conditions under which job insecurity is experienced, thereby enriching the quantitative findings. Together, these contributions provide a more complete theoretical account of how, when, and why job insecurity is associated with customer orientation.

## 2. Literature Review

To address the theoretical tension identified above, this study integrates four perspectives in a hierarchical rather than additive manner. The Job Demands–Resources (JD-R) model (Bakker & Demerouti, 2008) serves as the overarching architecture: job insecurity is modeled as a hindrance job demand, work engagement as the motivational pathway, customer orientation as the behavioral outcome, and industry tenure and gender as personal and structural resources that condition this process. Within this architecture, Stress Appraisal Theory (Lazarus & Folkman, 1984) performs the primary explanatory role at the appraisal stage, explaining why job insecurity is interpreted as a hindrance stressor that depletes psychological resources and reduces work engagement. Conservation of Resources (COR) theory (Hobfoll, 1989, 2011) performs the primary explanatory role at the contingency stage, explaining why employees with different resource reservoirs—such as those with longer industry tenure—are more or less able to withstand job insecurity. COR theory also explains the direct pathway: when job continuity is threatened, employees may conserve discretionary effort that is not formally required. Finally, Role Congruity Theory (Eagly & Karau, 2002) and Social Role Theory (Eagly, 1987) are not separate frameworks but extensions of the COR logic: they specify why women, on average, face greater structural resource constraints and caregiving demands (Muasya, 2016; Carli, 2020), unequal labor market opportunities (Blau & Kahn, 2017), and discrimination (Peng et al., 2024b), and are therefore more vulnerable when job insecurity is experienced (Shin et al., 2026). Thus, JD-R specifies the process, Stress Appraisal Theory specifies the appraisal mechanism, COR theory specifies resource contingencies, and role theories specify the structural basis of gender differences.

### 2.1. Job Insecurity and Customer Orientation

Job insecurity (JI) denotes a perceived threat to the continuity of employment, which may cause a feeling of fear and anxiety (Shoss, 2017). Job insecurity is categorized into two types: quantitative job insecurity and qualitative job insecurity (Hellgren et al., 1999). Quantitative job insecurity refers to the perceived threat to the continuity of employment itself, whereas qualitative job insecurity concerns threats to valued job features such as promotion, salary, and autonomy (Brondino et al., 2020). In the current tourism context, AI advancement and economic downturn primarily threaten employment continuity because many tourism roles are low-skilled, routinizable, and susceptible to automation or layoffs (Parvez et al., 2022). Accordingly, this study focuses on quantitative job insecurity.

Previous researchers have mixed opinions on the psychological and behavioral consequences of job insecurity. One stream of research enlightened by the Conservation of Resource Theory (Hobfoll, 2011) and impression management perspective (Shoss et al., 2023) believes that with potential job loss (resource), employees endeavor to safeguard their existing resources, such as their job, by allocating additional resources, such as time and effort, to create a favorable impression of their employers, by working harder (Hewlin et al., 2016). Another stream of studies rooted in the Stress Appraisal Theory (Lazarus & Folkman, 1984) posits that job insecurity is perceived by employees as a hindrance stressor (Currie et al., 2023) and thereby brings detrimental impacts on employee psychology and behavior, such as higher emotional exhaustion (Dogantekin et al., 2022), poorer mental health (Gupta & Dhar, 2024), decreased affective commitment (Haldorai et al., 2024), inferior service performance (He et al., 2023) and more withdrawal behaviors (Khan et al., 2022).

Customer orientation is defined as employee’s disposition to be thoughtful, helpful, cooperative, and considerate to customers (Hogan et al., 1984). Highly customer-oriented employees who have the most important traits to assure customer satisfaction (Hennig-Thurau, 2004) are treasures of tourism organizations (Liu et al., 2021). To sustain customer orientation, employees need psychological resources, such as the absence of emotional exhaustion (Choi et al., 2014) and the presence of affective commitment (Ribeiro et al., 2020). In other words, while job insecurity may drive tourism employees to seek opportunities for retention, the absence of psychological resources may pose challenges in maintaining customer orientation. This resource-depletion reasoning primarily describes an indirect process that functions through reduced motivational investment in work. A direct process may also operate after such motivational investment is taken into account: although job insecurity may motivate job-preserving effort, it does not necessarily extend to discretionary customer-oriented effort whose return is uncertain. COR theory suggests that employees protect remaining resources rather than invest them in efforts with uncertain returns (Hobfoll, 2011), and customer-oriented behavior often involves discretionary effort beyond basic service requirements (Hennig-Thurau, 2004). This conservation-of-effort process is conceptually distinct from resource depletion, even when employees remain motivated and engaged in their formal duties. Consistent with this reasoning and with prior tourism research (He et al., 2023; Dogantekin et al., 2022), we hypothesize:

**H1.**  *Job insecurity is negatively associated with customer orientation of tourism employees*.

### 2.2. The Mediating Role of Work Engagement

Given the mixed findings on the psychological and behavioral consequences of job insecurity, there might exist different pathways from job insecurity to employees’ psychological and behavioral consequences. As for the pathway from job insecurity to customer orientation, work engagement may serve as a mediator. Work engagement is a positive affective–motivational state of fulfillment characterized by vigor (physical strength of body and mind to give more effort to work), dedication (a feeling of enthusiasm, significance and challenge toward work) and absorption (full concentration on work) (Schaufeli et al., 2001). With high levels of energy, enthusiasm and concentration on work, engaged employees work more productively and efficiently (Vogel et al., 2022).

Work engagement has been widely studied in the framework of the Job Demand-Resources Model (JDR Model; Bakker & Demerouti, 2008). According to the JDR model, work engagement is an integrational outcome of many favorable psychological resources such as psychological capital (Paek et al., 2015), absence of emotional exhaustion (Ilies et al., 2020) and affective commitment (Poon, 2013). In addition, empirical research has reported the impairing effect of job insecurity on work engagement (Wang et al., 2015). Work engagement is also a strong predictor of many favorable psychological and behavioral consequences including customer orientation (Liu et al., 2021).

Drawing upon the integrated framework, we propose that work engagement serves as a key mediating mechanism. According to the JD-R model, job demands such as job insecurity consume employees’ energy and reduce their motivational resources, thereby lowering work engagement (Bakker & Demerouti, 2008). Stress Appraisal Theory further explains this process: when employees view job insecurity as a hindrance stressor, they experience anxiety and resource depletion, which undermines their vigor, dedication, and absorption in work (Lazarus & Folkman, 1984). COR theory adds that employees facing the threat of resource loss (i.e., job loss) will conserve remaining resources, making them less willing to invest energy in work engagement (Hobfoll, 1989). As work engagement is a well-established driver of customer orientation (Liu et al., 2021), reduced engagement resulting from job insecurity is expected to be associated with lower customer-oriented behaviors. An important clarification concerns why job insecurity is expected to reduce rather than increase work engagement, given the earlier discussion of impression-management and resource-preservation responses (Hewlin et al., 2016; Probst et al., 2007). We argue that these two responses operate through different mechanisms and target different outcomes. Impression-management behavior is a defensive, job-preserving strategy that may increase visible effort aimed at securing employment continuity; it does not, however, constitute the positive affective-motivational state of vigor, dedication, and absorption that defines work engagement. When job insecurity is appraised as a hindrance stressor, it generates anxiety and depletes psychological resources, thereby undermining the energetic and motivational core of engagement (Lazarus & Folkman, 1984). Thus, while job insecurity may stimulate short-term job-preserving effort, it is expected to reduce work engagement, which in turn lowers customer orientation. Thus, we hypothesize:

**H2.**  *Work engagement mediates the relationship between job insecurity and customer orientation*.

### 2.3. The Moderating Roles of Industry Tenure and Gender

The integrated framework also suggests that industry tenure moderates these relationships. According to COR theory, employees with longer industry tenure possess accumulated resources—including job-specific skills, professional networks, and established work routines—that help them withstand resource threats (Hobfoll, 1989). When facing job insecurity, these employees can draw upon their resource reservoirs to maintain their work engagement and customer orientation. Conversely, employees with shorter industry tenure lack these resources, making them more vulnerable to the negative effects of job insecurity (Karatepe & Karatepe, 2009). The JD-R model aligns with this reasoning: personal resources (such as industry tenure) buffer the negative impact of job demands on motivational outcomes (Bakker & Demerouti, 2008). In terms of the mediation sequence, industry tenure is theorized to moderate the first stage—the path from job insecurity to work engagement—because resource reservoirs most directly affect how employees respond motivationally to job insecurity. We also estimate moderation on the second stage (work engagement to customer orientation) to explore whether industry tenure additionally alters the translation of engagement into customer-oriented behavior. Therefore, we hypothesize:

**H3.**  *Industry tenure moderates the relationship between job insecurity and customer orientation such that the negative relationship is stronger for tourism employees with shorter industry tenure*.

**H4.**  *Industry tenure moderates the indirect relationship between job insecurity and customer orientation via work engagement, primarily by moderating the first stage of the mediation sequence (job insecurity to work engagement), such that the negative indirect relationship is stronger for tourism employees with shorter industry tenure*.

The gender hypotheses complement COR theory. Role Congruity Theory (Eagly & Karau, 2002) and Social Role Theory (Eagly, 1987) specify why gender is associated with differential resource availability and structural constraints. On average, women disproportionately bear family and caregiving responsibilities (Muasya, 2016; Carli, 2020), face unequal access to promotion and salary increases (Blau & Kahn, 2017), and experience higher exposure to workplace discrimination (Peng et al., 2024b). These structural disadvantages mean that when facing job insecurity, women tend to have fewer compensatory resources available, making them more susceptible to its negative effects (Shin et al., 2026). This pattern is particularly relevant in China, where women make up a large share of the tourism workforce but remain concentrated in lower-paid, lower-security frontline positions and are underrepresented in managerial roles (Peng et al., 2024b). These structural disadvantages are compounded by persistent caregiving demands that further constrain women’s available resources (Muasya, 2016; Carli, 2020). Within the JD-R architecture, gender operates as a structural condition shaping access to personal resources, while COR theory explains why this resource deficit increases vulnerability. These resource constraints may reduce work engagement (the indirect pathway) and may also directly reduce discretionary customer-oriented effort. In terms of the mediation sequence, gender is theorized to moderate the first stage—the path from job insecurity to work engagement—because structural resource constraints most directly affect how employees respond motivationally to job insecurity. We also estimate moderation on the second stage (work engagement to customer orientation) to explore whether gender additionally alters the translation of engagement into customer-oriented behavior. Based on this reasoning, we hypothesize:

**H5.**  *The negative direct relationship between job insecurity and customer orientation is stronger for female employees than for male employees*.

**H6.**  *Gender moderates the indirect relationship between job insecurity and customer orientation via work engagement, primarily by moderating the first stage of the mediation sequence (job insecurity to work engagement), such that the negative indirect relationship is stronger for female employees*.

**Figure 1:**
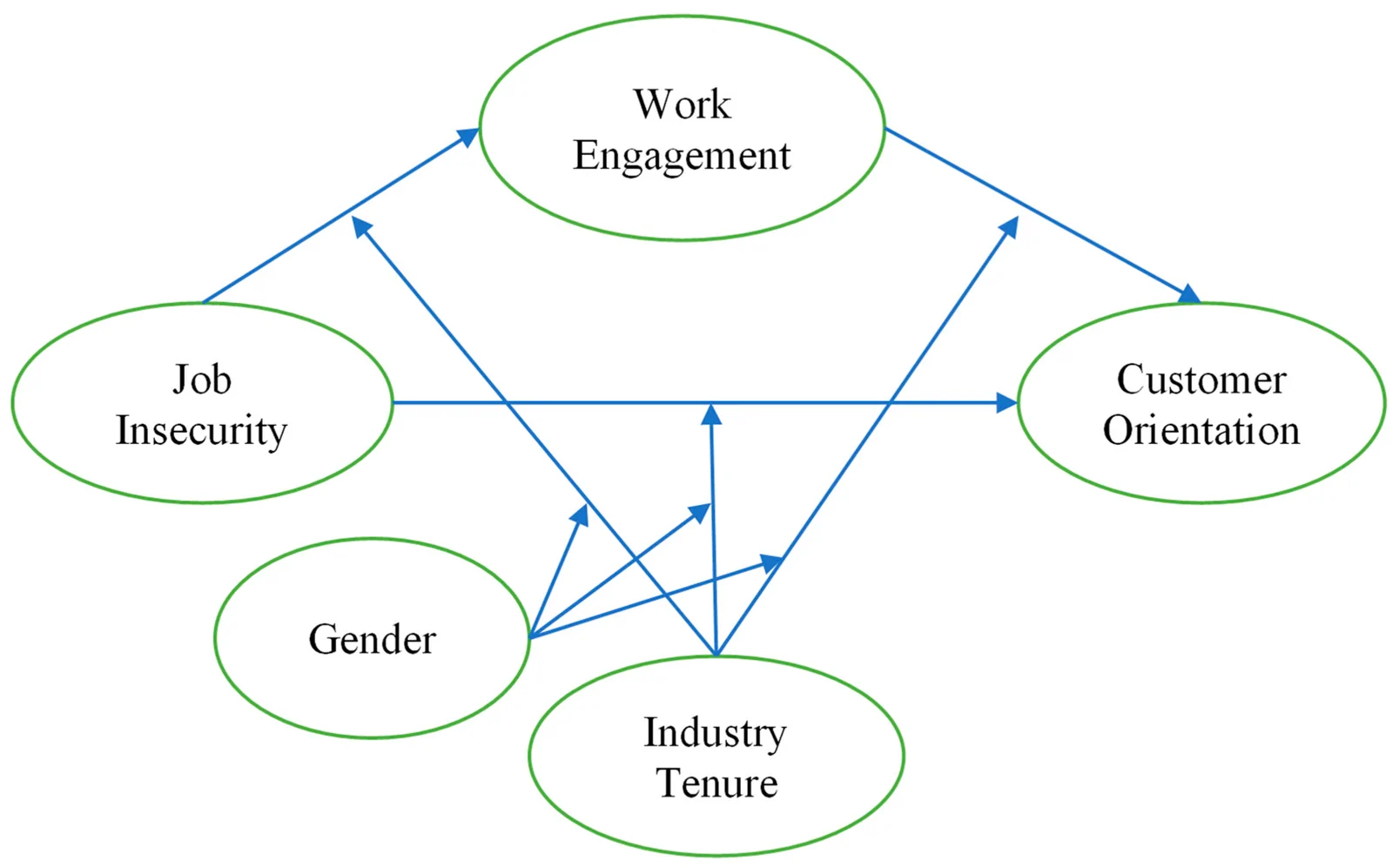
Hypothesized research model.

Figure 1 vividly shows the hypothesized model. 

## 3. Study 1: Quantitative Research

Study 1 empirically tested the hypotheses developed in the previous section. Specifically, it examined the pathway from job insecurity to customer orientation via work engagement, and the individual effects of gender and industry tenure on the pathway.

### 3.1. Methods

#### 3.1.1. Sample and Procedure

A two-wave survey with a time-lag of one month was conducted on full-time employees of four five-star hotels in a tourism city in China. China was selected as the study country because China is the second largest economy and has the fastest growth rate in the world (Muratova et al., 2019). Only five-star hotels were selected for the study, not only because five-star hotels represent the highest standard in the tourism industry, but because they play the key role in accommodating tourists in China as well (Qian & Law, 2021). The method of using a two-wave survey with one-month time-lag has been well recognized and adopted by scholars in human resource management for reducing common method variance and assuring temporal precedence (e.g., Lin et al., 2018; W. Zhou et al., 2016). The last four digits of participants’ cell phone number were used as identifiers to match the two sets of surveys.

Data collection occurred in two waves. Time 1 data were collected between April 8 and 23 April 2025. Time 2 data were collected between 8 May and 23 May 2025 (one month after Time 1). Regarding recruitment, with the assistance of the human resource departments of the four participating hotels, an email containing a link to the online questionnaire was sent to all full-time employees via the hotels’ internal email systems. Participation was voluntary, and employees were informed that their responses would be used for research purposes only and would remain confidential.

Prior to participation, all respondents provided informed consent after receiving a detailed explanation of the study’s purpose, procedures, confidentiality measures, and their right to withdraw at any time without consequence. For participants aged 17 (N = 4), parental or guardian consent was obtained in addition to participant assent, and all were legally employed in accordance with Chinese labor laws. At Time 1, a total of 426 participants completed the questionnaires that collected demographic (age, education, marital status, and managerial vs. non-managerial position), job insecurity, industry tenure, and gender information. Marital status was collected for descriptive purposes but was not included in the focal hypothesis-testing models because preliminary analyses showed no significant association with the mediator or the outcome, and excluding it preserved model parsimony. At Time 2, respondents of Time 1 were invited to answer questionnaires measuring work engagement and customer orientation. A total of 211 respondents completed the Time 2 survey. Of the 426 participants who completed the Time 1 survey, 211 completed the Time 2 survey, yielding a response rate of 49.5%. The survey platform was programmed to require completion of all items before submission, ensuring no item-level missing data among participants in either wave. Because reasons for non-response were not systematically recorded, we cannot confirm the specific causes of attrition; however, independent-samples t-tests and chi-square tests showed no significant differences between completers and non-completers in age, gender, education, marital status, position type, industry tenure, or Time 1 job insecurity. The Process Macro analysis technique (Hayes, 2017) was employed to test the hypotheses.

To assess whether attrition introduced systematic bias, we compared respondents who completed both waves (N = 211) with those who dropped out after Time 1 (N = 215) in terms of all demographic variables and Time 1 job insecurity. Independent samples t-tests and chi-square tests revealed no significant differences between the two groups in age (t(424) = 0.87, *p* = 0.38), gender (χ^2^(1) = 0.32, *p* = 0.57), education (χ^2^(4) = 1.96, *p* = 0.74), marital status (χ^2^(2) = 0.54, *p* = 0.76), position type (χ^2^(1) = 0.21, *p* = 0.65), industry tenure (t(424) = −0.64, *p* = 0.52), or job insecurity (t(424) = 0.73, *p* = 0.47). These results suggest that attrition was largely random and unlikely to bias the findings.

A post hoc power analysis using G*Power 3.1 (Faul et al., 2009) indicated that with 211 respondents and up to 15 predictors in the regression model, the study had 80% power to detect a medium effect size (f^2^ = 0.09) at α = 0.05, which is adequate for testing the hypothesized moderated mediation model. This level of power is considered acceptable for detecting the focal three-way interaction in a regression framework (Aiken & West, 1991).

Employees were nested within four hotels. Because the number of clusters is very small (k = 4), including hotel fixed effects would substantially reduce the degrees of freedom and produce unstable estimates, and cluster-robust standard errors are not reliable with so few clusters. We therefore did not include hotel indicators in the focal models. This limitation is acknowledged in Section 5.3, and future research with a larger number of hotels should employ multilevel modeling to account for within-cluster correlation.

#### 3.1.2. Measures

All scales were translated using the back-translation procedure (Brislin, 1980) and had been previously validated in Chinese samples; no items were adapted.

Job insecurity was measured using De Witte’s (2000) four-item scale assessing quantitative job insecurity (threat to employment continuity). Sample item: “I fear that I might lose my job.” Responses used a 7-point Likert scale (1 = strongly disagree to 7 = strongly agree). It has a Cronbach’s α = 0.94, CR = 0.94, and AVE = 0.80. The Chinese version has been validated in Chinese organizational contexts (Wang et al., 2015).

Work engagement was measured using the three items of the short version of the Utrecht Work Engagement Scale (Schaufeli et al., 2017): vigor, dedication, and absorption. Sample item: “At my work, I feel bursting with energy”. The scale uses a 7-point Likert scale. It has a Cronbach’s α = 0.92, CR = 0.92, and AVE = 0.72. The Chinese version has been validated (Fong & Ng, 2012).

Customer orientation was measured using Donavan et al.’s (2004) 13-item scale. Sample item: “I enjoy taking care of my customers.” The scale uses a 7-point Likert scale. It has a Cronbach’s α = 0.97, CR = 0.97, and AVE = 0.83. The Chinese version has been validated in hospitality contexts (Liu et al., 2021).

Industry tenure was measured in years. Control variables included age, education, and position type. Gender and industry tenure were included as moderators, and their main effects were also estimated in the models. Marital status was not included in the focal analyses for the reason explained in Section 3.1.1.

#### 3.1.3. Analytical Strategy

The hypothesized moderated mediation model was tested using the PROCESS Macro for SPSS (Version 3.5, Model 73) (Hayes, 2017) with 5000 bootstrap resamples and 95% bias-corrected confidence intervals. This model is appropriate for testing H2 (mediation), H3–H4 (moderation by industry tenure), and H5–H6 (moderation by gender). Gender was coded as 0 = female and 1 = male. This coding is used consistently in all interaction terms and conditional-effect analyses. The model estimates moderation of both the job insecurity → work engagement path (first stage) and the work engagement → customer orientation path (second stage), allowing the hypothesized first-stage moderation to be tested directly.

Continuous variables (job insecurity, work engagement, industry tenure) were mean-centered prior to analysis to reduce multicollinearity and facilitate interpretation of interaction terms (Aiken & West, 1991). Industry tenure was measured in years as a continuous variable. The conditional indirect effects of job insecurity on customer orientation via work engagement were estimated at three levels of industry tenure: one standard deviation below the mean (M−1SD), at the mean (M), and one standard deviation above the mean (M+1SD), which were calculated separately for male and female employees. The index of moderated mediation (Hayes, 2015) was computed to formally test whether the indirect effect differed significantly across levels of the moderators. Statistical significance was determined based on whether the 95% bias-corrected bootstrap confidence interval did not include zero.

### 3.2. Results

#### 3.2.1. Preliminary Analyses

Preliminary analyses included the common method bias test and confirmatory factor analysis. To reduce the risk of common method bias, several procedural remedies were implemented (Podsakoff et al., 2003). First, temporal separation was introduced between the measurement of the predictor variable (job insecurity, Time 1) and the outcome variables (work engagement and customer orientation, Time 2), with a one-month time lag. Second, response anonymity was ensured to reduce evaluation apprehension. Third, item blocks were visually separated in the questionnaire.

As a supplementary check, Harman’s single-factor test was performed. The extracted first principal component explained 43.06% of the total variance, below the conventional 50% threshold. However, given that this test has significant limitations as a diagnostic tool for common method bias (Howard et al., 2024), this result should be interpreted with caution. We address this limitation further in Section 5.3.

Confirmatory factor analysis on the three latent variables of this study tests the discriminant validity between variables. The fitting indices of single-factor model, two-factor models and three-factor model are shown in Table 1. The goodness of fit between the observed data and three-factor model is the best. Both the chi-square test and the model fit index show that there is a significant difference between the three-factor model and the alternative model, justifying the discriminant validity of the data.

#### 3.2.2. Descriptive Analysis

Table 2 shows the demographic characteristics of the research sample. Table 3 shows the mean, standard deviation and correlation coefficient between the study variables. The sample consisted of 211 employees. Female employees constituted 55.5% and male employees 44.5%, meaning that there were 11 percent more female than male respondents. The largest age groups were 18–30 years (48.8%) and 31–50 years (46.0%), followed by 51 years and older (3.3%) and 17 years and younger (1.9%). Educational attainment was concentrated at the college level (64.5%), followed by middle school (26.1%), high school (7.1%), graduate degree (1.9%), and some school (0.5%). Most respondents were in non-managerial positions (59.2%), while 40.8% held managerial positions. Regarding industry tenure, 47.4% of respondents had less than three years of tenure, and 43.1% had more than five years. The correlation coefficient in Table 3 reveals that job insecurity is negatively related to both work engagement and customer orientation, and work engagement is positively related to customer orientation.

#### 3.2.3. Hypothesis Testing

Table 4 demonstrates the results of the hypothesis testing. The results of the dependent variable model show that job insecurity is significantly and negatively associated with customer orientation (B = −0.44, *p* < 0.001), supporting H1. As for the results of the mediator variable model, job insecurity is significantly and negatively associated with work engagement (B = −0.51, *p* < 0.001). Meanwhile, the association between work engagement and customer orientation in the dependent variable model is significantly positive (B = 0.51, *p* < 0.001), indicating that work engagement plays a significant mediating role between job insecurity and customer orientation. H2 is supported. To formally test the mediation hypothesis (H2), we examined the indirect effect of job insecurity on customer orientation through work engagement. The indirect effect was B = −0.26, 95% CI [−0.44, −0.10], which does not include zero, indicating that work engagement mediates the relationship between job insecurity and customer orientation. The direct association between job insecurity and customer orientation, after accounting for the mediator, remained significant (B = −0.44, *p* < 0.001), indicating partial mediation. The dependent variable model is significant overall (R^2^ = 0.61, *p* < 0.001). The R^2^ change is also significant after adding the interaction term of job insecurity × industry tenure × gender, indicating that industry tenure and gender can further moderate the relationship between job insecurity and customer orientation. H3 was tested by analyzing the job insecurity × industry tenure interaction. H5 was tested by analyzing the job insecurity × gender interaction. The three-way job insecurity × industry tenure × gender interaction indicates whether the moderating role of industry tenure itself differs by gender. Because the three-way interaction was significant, we interpret H3 and H5 based on the conditional-effect pattern. Simple slope analyses showed that the negative direct association was significant only for female employees with shorter industry tenure. Although the three-way interaction was statistically significant, its incremental contribution was small (R^2^ change = 0.01). In the mediator variable model, the two-way interaction of job insecurity × industry tenure on work engagement is not significant (B = 0.03, *p* = 0.07). In the dependent variable mode, the two-way interaction of work engagement × industry tenure is not significant either (B = −0.01, *p* = 0.66). These indices mean that industry tenure does not moderate the indirect relationship between job insecurity and customer orientation via work engagement. That is to say, H4 is not supported. Similarly, the effect of the three-way interaction of job insecurity × industry tenure × gender on work engagement in the mediator variable model is not significant (B= −0.03, *p* = 0.20), and the effect of the three-way interaction of work engagement × industry tenure × gender on customer orientation in the dependent variable model is not significant either (B= 0.03, *p* = 0.31). These indices show that there is no gender difference in the non-significant moderating role of industry tenure in the indirect relationship between job insecurity and customer orientation via work engagement. Thus, H6 is not supported. To formally test the moderated mediation hypotheses (H4 and H6), we computed the conditional indirect effects of job insecurity on customer orientation via work engagement at different levels of industry tenure, calculated separately for male and female employees, along with their 95% bias-corrected bootstrap confidence intervals. As shown in Table 5, the conditional indirect effects of job insecurity on customer orientation via work engagement were not significant in any study subgroup (all 95% bias-corrected bootstrap confidence intervals included zero). The index of moderated mediation for the three-way interaction was 0.01, with a 95% CI of [−0.01, 0.04], which includes zero, further indicating that the moderated mediation effects were not statistically significant (Hayes, 2015). These results are consistent with the non-significant interaction terms reported in Table 4 and indicate that H4 and H6 are not supported.

**Figure 2:**
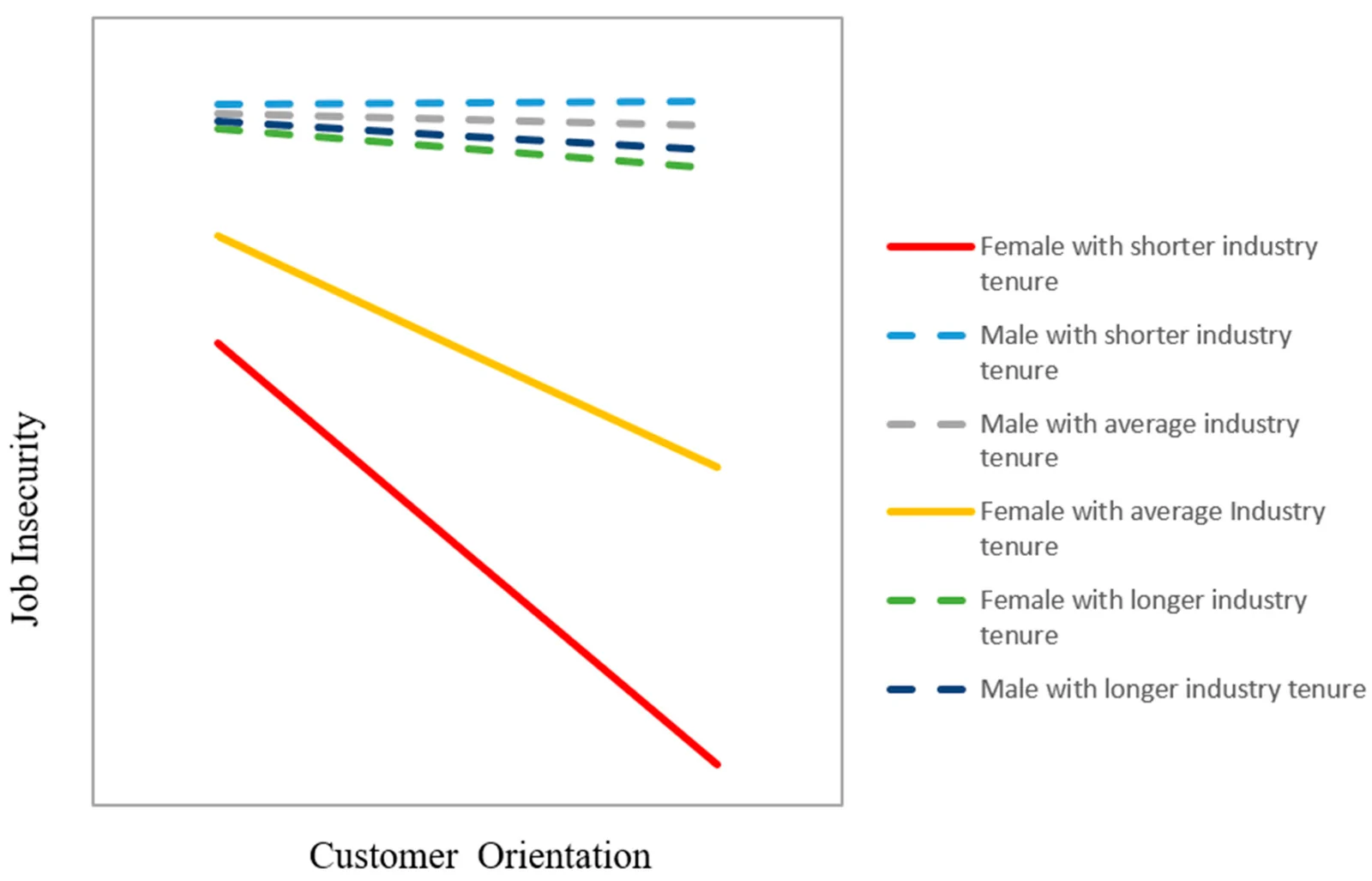
The direct associations between job insecurity and customer orientation across gender and industry tenure groups.

Table 5 further shows the direct relationship between job insecurity and customer orientation of male and female employees with different levels of industry tenure. The negative relationship between job insecurity and customer orientation is only significant for female employees with industry tenure at or below the mean level, but not significant for other types of employees. As female employees’ industry tenure increases, the negative association between job insecurity and customer orientation decreases. Figure 2 vividly shows the moderating roles. The solid lines present the significant role of job insecurity on customer orientation, and the dotted lines present the non-significant roles.

## 4. Study 2: Qualitative Research

The results of the quantitative study can answer the “how” and “when” questions by numerically demonstrating the study relationships. However, they offer insufficient insight into the “why” question that seeks to reveal deeper plausible reasons for the phenomenon. Therefore, a qualitative study (Study 2) is needed to clarify and elaborate the results from the quantitative research (Study 1).

### 4.1. Methods

The interview protocol (Table 6) focused on quantitative job insecurity—concerns about job loss and employment continuity—to maintain conceptual consistency with the quantitative measure (De Witte, 2000). Although the protocol focused on quantitative job insecurity, one interview question also referred to career development opportunities. This question was retained to allow participants to express general career concerns. In the analysis, responses concerning career development were coded separately as qualitative job insecurity and were not used to elaborate the quantitative findings unless participants explicitly linked them to employment continuity. This ensures conceptual alignment with the De Witte (2000) measure used in Study 1. Semi-structured interviews were conducted to obtain additional qualitative information about the relationship between job insecurity and customer orientation, and to find potential explanations for the results from the quantitative study.

(1) Participant selection and sampling

Participants (Table 7) were selected through convenience sampling from the same four five-star hotels that participated in Study 1. None of the qualitative interviewees had participated in the quantitative survey, ensuring that the qualitative data provided independent perspectives rather than follow-up explanations from the same respondents. While sampling was not purposively pre-selected based on gender and tenure, the final sample (N = 12) included an equal gender distribution (six male, six female) and a range of industry tenures (1 to 15 years), reflecting the diversity of the hotel workforce.

(2) Interview procedures

Prior to each interview, participants provided informed consent after receiving an explanation of the study’s purpose, the voluntary nature of their participation, their right to withdraw at any time, and the confidentiality measures in place. All participants provided verbal consent before the interviews began. The interviews were conducted online by two researchers, both native Mandarin speakers with prior experience in qualitative interviewing. Each interview lasted 30 to 45 min and was audio-recorded with the participants’ permission. All interviews were conducted in Chinese to allow participants to express themselves naturally and comfortably.

(3) Translation process

All interviews were transcribed verbatim in Chinese. Following the back-translation approach (Brislin, 1980), the Chinese transcripts were translated into English by one bilingual researcher and then back-translated into Chinese by a second bilingual researcher. Discrepancies were discussed and resolved to ensure accuracy and preserve the original meaning of the participants’ statements. The quotations presented in this manuscript represent the English translations of the participants’ original Chinese statements.

(4) Data saturation

The number of interviews (N = 12) was determined based on the principle of data saturation (Guest et al., 2006). After each interview, the two researchers independently reviewed the transcript and discussed emerging themes. A saturation log was maintained, documenting when new codes ceased to emerge and when existing themes were well-developed. Saturation was considered achieved when three consecutive interviews (interviews 10, 11, and 12) yielded no new codes and all core themes were sufficiently developed (Guest et al., 2006). This systematic approach goes beyond simply noting that the final three interviews produced no new information.

(5) Coding approach and analysis

The analysis followed Braun and Clarke’s (2006) six-phase framework: ① familiarization with the data through repeated reading of transcripts; ② generation of initial codes; ③ search for themes by collating codes; ④ review of themes; ⑤ definition and naming of themes; and ⑥ production of the report. The coding employed both deductive and inductive approaches; deductive codes were derived from the hypothesized relationships (e.g., “job insecurity as a hindrance stressor” and “work engagement as a mediating mechanism”), while inductive codes emerged from the data without preconceptions (e.g., “labor shortage as a buffer” and “AI not perceived as a threat”).

(6) Credibility and reflexivity

To enhance trustworthiness and credibility, the two researchers independently coded the first three transcripts, compared their coding schemes, resolved discrepancies through discussion, and then coded the remaining transcripts using the agreed-upon framework (Lincoln & Guba, 1985). Both researchers maintained reflexive journals to document their assumptions, potential biases, and decision-making processes throughout the analysis (Olmos-Vega et al., 2023). Peer debriefing was conducted through regular discussion meetings at each stage of the analysis to challenge interpretations and reduce individual bias. Additionally, member checking was performed: key findings were reviewed with two participants (P5 and P8), who confirmed that our interpretations accurately reflected their experiences.

To further minimize confirmation bias, the two researchers actively sought alternative interpretations and contradictory accounts throughout the coding process. Cases where participant responses did not align with the hypothesized relationships were explicitly discussed. For example, while most interviewees described job insecurity as detrimental to their work engagement, several participants expressed little concern about job security (see Section 4.2.3). The researchers also considered alternative explanations—for instance, whether reduced customer orientation might reflect general job dissatisfaction rather than job insecurity specifically—and concluded that the evidence more strongly supported the hypothesized mechanisms. This systematic consideration of alternative interpretations enhances the credibility of the qualitative findings (Lincoln & Guba, 1985). Table 8 shows the thematic coding structure that emerged from the interview analysis.

### 4.2. Results

The qualitative phase was designed to elaborate the quantitative findings by addressing two core “why” questions: (1) why does job insecurity reduce customer orientation? and (2) why is this effect stronger for female employees with shorter industry tenure? The interviews provide contextual explanations for these questions rather than tests of the hypotheses. The qualitative evidence should therefore be read as illustrative and exploratory, not as hypothesis verification.

#### 4.2.1. Explanations Consistent with the Quantitative Findings

Regarding why job insecurity reduces customer orientation, participants described both a direct conservation-of-effort process and an indirect process through reduced work engagement. Following Venkatesh et al.’s (2016) guidelines for mixed-methods integration, the qualitative findings are used to elaborate upon and provide explanations consistent with the quantitative results. Rather than simply confirming the statistical relationships, the interviews offered rich contextual insights into why and under what conditions these associations manifest. Below, we present four sets of explanations aligned with the hypothesized relationships, followed by an analysis of negative cases that diverged from the dominant pattern in Section 4.2.3.

(1) Participants described how job insecurity reduced their willingness to invest discretionary effort in customer-oriented behavior.

“I understand that being customer orientated is my obligation, but to be honest, I think my feeling of job insecurity could impair my effort of customer orientation. Whenever I think of my unsure future career, I have to wonder why I have to step forward to help my customers.”

(P1)

“I believe feeling insecure on my job would affect my service quality. If this job is stable, I of course would like to provide extra service to my customers.”

(P2)

“I would like to provide extra service to nice and respectful customers. It is no problem at all for me to provide basic service to those rude customers, but very hard to keep oriented to them. After all, this job is not a ‘golden bowl’.”

(P3)

(2) Participants also described how job insecurity reduced their concentration and absorption in work, which made it harder to maintain customer orientation.

“Whenever I think of my unsure future career, I have to wonder why I have to step forward to help my customers with extra service.”

(P1)

“When you think of your unsure future career, you have to think about what other career opportunities you may have, and then you cannot focus on what you are doing. How could you stay fully customer-oriented, when you don’t concentrate on your job?”

(P2)

“I would agree that I would not be able to be as absorbed in my work as usual when I worry about the work and promotion opportunities. I could do better when I am more engaged in what I am doing.”

(P5)

(3) Participants’ accounts help explain why the direct association was concentrated among female employees with a tenure at or below the mean. Longer-tenured employees described customer-oriented service as habitual.

“I have been working in this industry for 7 years, and trying to provide customer-oriented service has become part of my habits. I do not think a habit can be easily influenced by a feeling of job insecurity.”

(P5)

“My job is not a stable position, but it is easy to find such a position in the market. If I lost this job, I can find one somewhere else. I am not sure I feel secure or insecure about my job, but feelings of insecurity or security would not affect my work quality.”

(P6)

“I can see the job security in this industry is threatened by many factors, including technology, economy, and etc., but as a HR manager, I am supposed to be rational instead of emotional. Not letting personal feelings impair your work is a basic professional quality.”

(P11)

(4) Participants’ accounts also illustrate contextual and coping differences that help interpret why the direct association was stronger for female employees with a tenure at or below the mean.

“Job security in this industry is not under my control. What I can control is my work performance. What I can only do to secure my job and career opportunities is to work harder rather than to worry”

(P7)

“I am just too busy to worry about job insecurity. I have to do multiple tasks every day and try to provide excellent service in every single task. I interact with customers face-to-face; this kind of position gets complaints pretty easy.”

(P8)

#### 4.2.2. Plausible Reasons for the Identified Relationships

To understand why the direct effect is stronger for female employees with shorter industry tenure, participants described how industry tenure and gender shape their responses to job insecurity.

(1) Under job insecurity, some employees are reluctant to maintain customer orientation because job insecurity discounts the return on their customer-oriented behaviors.

“Whenever I think of my unsure future career, I have to wonder why I have to step forward to help my customers…When people believe their hard working can get them something they want, they are likely to work hard. But if they are not sure whether they can get what they want with some kind of hard working, they probably would not like to put their effort in.”

(P1)

“If this job is stable, I of course would like to provide extra service to my customers…With this unstable position type, providing extra service to customers cannot guarantee anything for me.”

(P2)

(2) Participants described reduced concentration and absorption as a process linking job insecurity to lower customer orientation. This helps interpret the quantitative mediation finding.

“When you think of your unsure future career, you have to think about what other career opportunities you may have, and then you cannot focus on what you are doing. How could you stay fully customer-oriented, when you don’t concentrate on your job?”

(P2)

“I would agree that I would not be able to be as absorbed in my work as usual when I worry about the work and promotion opportunities. I could do better when I am more engaged in what I am doing.”

(P5)

(3) To understand why the direct effect is stronger for female employees with shorter industry tenure, participants described how industry tenure and gender shape their responses to job insecurity.

“I have been working in this industry for 7 years, and trying to provide customer-oriented service has become part of my habits. I do not think a habit can be easily influenced by a feeling of job insecurity.”

(P5)

“I am required to provide customer-oriented service. I have been used to do it.”

(P12)

“I understand that being customer orientated is my obligation, but to be honest, I think my feeling of job insecurity could impair my effort of customer orientation…I have to be reminded if I need to provide any extra service.”

(P1)

(4) Participants described how gender shaped structural constraints and contextual demands shaped their coping responses to job insecurity.

“I am just too busy to worry about job insecurity. I have to do multiple tasks every day and try to provide excellent service in every single task. I interact with customers face-to-face; this kind of position gets complains pretty easy.”

(P8)

“It is not easy to get rid of this feeling of job insecurity which often distracts me from my work.

(P1)

#### 4.2.3. Negative Cases and Divergent Perspectives

While most interviewees reported experiencing job insecurity and its detrimental effects, a subset of participants (P4, P6, and P9) expressed little or no concern about job security. These negative cases—instances that did not conform to the hypothesized pattern—provide valuable boundary conditions for understanding when job insecurity does not arise.

Two main reasons emerged for the absence of job insecurity concerns. First, several participants cited the ongoing labor shortage in the tourism industry as a source of employment stability:

“In the tourism industry, employees are not the party who need to worry about security, but the employers are in the place of insecurity. I mean employers worry about unsecured labor force.”

(P4)

“My job is not a stable position, but it is easy to find such a position. If I lost this job, I can find one somewhere else pretty soon.”

(P6)

Notably, AI-related concerns emerged inductively from the data, as the interview protocol did not specifically prompt participants to discuss technological replacement. Several participants spontaneously raised this topic when discussing their perceptions of job security. Their responses revealed a range of perspectives:

The second reason for the absence of job insecurity concerns was that some participants perceived AI technologies as insufficiently advanced to replace human workers:

“I do not think my job can be replaced by machines in the near future… Yes, technologies develop fast, but as you see, we only have robots deliver meals… Robots may be able to do more, but that is not going to happen soon.”

(P9)

These divergent perspectives suggest that job insecurity is not universal among tourism employees. Labor shortages and the perception that AI is unlikely to replace a particular job are contextual antecedents that shape whether employees experience job insecurity in the first place, rather than boundary conditions of the job insecurity–customer orientation association. An additional inductively derived pattern in this small qualitative sample is that employees who are more committed to the industry as a long-term career may be more likely to experience job insecurity. This pattern is presented as a tentative, inductively derived proposition rather than a general boundary condition. Because industry commitment was not measured quantitatively and the qualitative sample was small (N = 12), future research is needed to test whether commitment strengthens job-insecurity perception.

#### 4.2.4. An Exploratory Proposition: The Paradox of Commitment

Mixed research methods can also reveal valuable complementary information that might have been overlooked by a single quantitative study (Venkatesh et al., 2016). Through analyzing the transcripts, an additional pattern emerged: employees who are more committed to the tourism industry reported experiencing stronger job insecurity.

“I can see that some tasks in the industry have been and will be replaced by technologies. I do not want my job to be replaced by technologies, after all I love my job… I often think about what I can do to be not replaced by robots.”

(P5)

“Some of my tasks have been replaced by robots. You see, it was my responsibility to deliver meals, now it is done by robots. I believe robots will be able to do more of my tasks, so I like rotation opportunities to learn more skills… Yes, I want to stay in this industry as my life-long career.”

(P7)

These accounts suggest a possible paradox: some employees who expressed strong commitment to the tourism industry also described heightened job insecurity. Because this pattern is based on a small number of interviews and industry commitment was not measured in the quantitative study, we present it as an exploratory, inductively derived proposition rather than a generalized finding. Future research should include industry commitment as a measured variable and test whether commitment strengthens job-insecurity perceptions.

## 5. Discussion and Conclusions

In order to comprehend how, when and why job insecurity is associated with the customer orientation of tourism employees and to enhance management strategies for motivating them amidst job insecurity, this study explored the direct and indirect relationship between job insecurity and customer orientation. The research first tested a moderated mediation model integrating work engagement acting as the mediator and industry tenure and gender serving as moderators, and then explored plausible reasons for the identified relationships. The results reveal that job insecurity is associated with lower customer orientation, particularly through work disengagement. There are no individual differences in the indirect pathway from job insecurity to work engagement and then to customer orientation. However, the direct association between job insecurity and customer orientation is only significant for female tourism employees with shorter industry tenure. The results further revealed the reasons for the phenomenon and additionally discovered that not all the tourism employees suffer from job insecurity, but those who are more committed in the tourism industry are more likely to worry. These findings yield both theoretical and practical implications.

### 5.1. Theoretical Implications

First, given the controversial arguments on the psychological and behavioral consequences of job insecurity, the results of this study provide empirical evidence for the impairing effect of job insecurity. In line with the Stress Appraisal Theory and its related literature (Dogantekin et al., 2022; Haldorai et al., 2024), the results of the present study justify that job insecurity may be perceived as a hindrance stressor by tourism employees, thereby generally eroding their work engagement and customer orientation. The qualitative results further explain that under job insecurity, customer-oriented service would not bring expected return to employees; therefore, they may hesitate to provide customer-oriented service. These findings urge future researchers and tourism practitioners to develop management strategies to mitigate the negative impact of job insecurity.

Second, the results also reveal two different pathways from job insecurity to customer orientation, both direct and indirect. These results apply the JD-R logic to the job insecurity–customer orientation relationship and illustrate how the JD-R framework can accommodate both direct and indirect pathways in this context. While job insecurity might drive individuals to seek opportunities for retention, the fear and anxiety associated with the possibility of job loss can hinder their active involvement in work. Consequently, this work disengagement may undermine their customer orientation. These findings encourage tourism researchers and practitioners to develop management strategies to maintain the work engagement of tourism employees and ensure their customer orientation.

Third, the results further demonstrate individual differences in the direct and indirect pathways from job insecurity to customer orientation. The overall indirect effect of job insecurity on customer orientation through work engagement was significant at the sample level (H2). However, the conditional indirect effects did not vary significantly by industry tenure or gender, and the indices of moderated mediation for H4 and H6 were not significant. Therefore, work engagement should be interpreted as an overall sample-level mediating mechanism, not as a universal mechanism that operates equally in all subgroups. The non-significant conditional indirect effects and the non-significant index of moderated mediation may reflect limited statistical power for detecting moderated mediation. Because the indirect effect is a product of two paths, testing whether it varies across levels of a moderator requires greater power than when testing a single interaction effect (Hayes, 2015). The first-stage interaction for gender, although significant, did not translate into a significant conditional indirect effect or index of moderated mediation.

However, the direct pathway showed a more complex pattern. Although the three-way interaction (job insecurity × industry tenure × gender) was statistically significant, its incremental contribution was small (R^2^ change = 0.01). The simple slope analysis revealed that the negative direct association was significant only for female employees with shorter industry tenure. Consistent with recommendations for interpreting interaction effects (Aiken & West, 1991), we interpret this as suggestive evidence of subgroup differences rather than definitive evidence of effects existing in one group but not another, particularly given the small effect size.

We interpret this pattern through structural and contextual mechanisms rather than dispositional traits. Junior female employees face compounded stressors at the intersection of career stage and gender: they have fewer accumulated resources (professional networks and job-specific skills) to buffer job insecurity (Karatepe & Karatepe, 2009; Hobfoll, 1989) while simultaneously experiencing systemic labor market disadvantages—unequal access to promotions, occupational segregation in low-security service roles, and workplace discrimination—that amplify the perceived threat of job loss (Blau & Kahn, 2017; Wolfram et al., 2020; Peng et al., 2024b). In addition, women’s disproportionate caregiving responsibilities leave fewer available resources to cope with workplace stressors (Muasya, 2016; Carli, 2020). These structural disadvantages help explain why this subgroup is particularly susceptible to the direct negative association between job insecurity and customer orientation, aligning with research suggesting that women’s greater vulnerability reflects systemic inequalities rather than dispositional factors (Shin et al., 2026).

It is important to clarify that the conclusion regarding gender differences is based on the quantitative findings (N = 211), where the three-way interaction (job insecurity × industry tenure × gender) was statistically significant (B = −0.04, *p* = 0.02). The qualitative interviews (N = 12) provided illustrative examples that were consistent with this quantitative pattern, but the small qualitative sample should not be interpreted as independently establishing gender differences. Rather, the qualitative data offered contextual explanations that help interpret the quantitative results. These explanations should be viewed as preliminary insights requiring further investigation with larger, more diverse qualitative samples.

These insights lay the groundwork for future research and for developing varied management strategies to motivate employees amidst job insecurity.

Fourth, the qualitative results provided complementary insights regarding who are more likely to have the feeling of job insecurity. Given the labor shortage in the tourism industry, some employees do not worry about job insecurity as they may intend to leave. However, the others who suffer from job insecurity are those who are more committed to the tourism industry and worry about their long-term career. This exploratory and inductively derived pattern tentatively suggests that employees who are more committed to the tourism industry may be more likely to experience job insecurity. This finding urges both academic researchers and industry practitioners to pay extra attention to job insecurity of tourism employees, as employees feeling job insecurity are the stable labor force of the industry and need to be cherished. Because the qualitative sample was small and industry commitment was not measured quantitatively, this should not be treated as a generalized finding. Future research should measure industry commitment and test whether it strengthens job-insecurity perceptions.

Fifth, the explanatory sequential design enables integration of the quantitative and qualitative findings. The qualitative data elaborated upon the three quantitative patterns by revealing the underlying mechanisms: reduced work engagement reflects perceived diminished returns on effort; junior female employees’ direct vulnerability reflects compounded structural disadvantages; and industry tenure buffers through habits and career mobility rather than through engagement protection. More importantly, the qualitative data qualified the quantitative results by revealing that job insecurity is not universal—several participants expressed no concern about job security (see Section 4.2.3). This suggests that job insecurity is particularly salient among employees committed to the industry as a long-term career, whereas those who view their job as interchangeable or who perceive technological replacement as distant may remain unaffected. This inductive insight refines the quantitative conclusions by specifying the boundary conditions under which job insecurity does and does not arise.

### 5.2. Practical Implications

Primarily, the study findings serve as a reminder to tourism managers not to unquestioningly assume that employees’ job insecurity will enhance their customer orientation or other aspects of job performance; in reality, the opposite may hold true. Tourism managers need to find ways to lower employees’ job insecurity and its negative impact on employee psychology and behaviors. Although the advancement of AI technologies has greatly improved the service efficiency of the tourism industry, AIs are far inferior to humans in providing “warm services”. Tourism managers can alleviate employees’ job insecurity by emphasizing the advantages of humans in “warm services” and incentivize such services so that employees can be more optimistic and committed to providing high-quality and warm services.

Second, for the majority of tourism employees, job insecurity affects their customer orientation by reducing their work engagement level. Therefore, tourism managers need to pay attention to employees’ work engagement level, and ensure employees’ work engagement by providing sufficient organizational resources, such as training, empowerment, organizational support, autonomy, etc., to prevent the decrease in customer orientation under job insecurity. These organizational resources have been suggested by prior research and were not directly examined as interventions in this study.

Last but not least, our findings suggest that female employees with shorter industry tenure may be a particularly vulnerable group. This pattern is consistent with prior research indicating that career adaptability facilitates job performance among junior tourism employees (Peng et al., 2024a) and that organizational support plays a significant role in employee retention and performance (X. Zhou et al., 2022). While our study did not directly evaluate specific interventions, prior literature suggests several possible organizational responses that could be considered to support this subgroup. These may include cultivating a family-friendly workplace, providing mentorship and feedback, and offering more opportunities to voice concerns and participate in decision-making (Peng et al., 2024a; X. Zhou et al., 2022). However, these suggestions are informed by the broader literature and should be empirically evaluated in future research before being implemented as formal organizational policies. We encourage future studies to test the effectiveness of such interventions in mitigating the adverse effects of job insecurity on customer orientation among junior female employees.

### 5.3. Limitations and Future Directions

Although this study has multiple theoretical and practical contributions, it is not without limitations.

First, the quantitative sample experienced approximately 50% attrition between the two survey waves (from 426 at Time 1 to 211 at Time 2). While attrition analysis suggested random patterns (see Section 3.1.1), some degree of bias cannot be completely ruled out.

Second, although the two-wave design with a one-month time lag helps establish temporal precedence between job insecurity (Time 1) and the outcomes (Time 2), work engagement and customer orientation were measured concurrently at Time 2. Therefore, while the findings are consistent with a mediation model, definitive causal claims cannot be made.

Third, despite procedural remedies including temporal separation, anonymity, and visual separation of item blocks, work engagement and customer orientation were both self-reported at Time 2, meaning same-source variance cannot be completely ruled out.

Fourth, although a post hoc power analysis indicated adequate power to detect a medium effect size, the statistical power for detecting the focal three-way interaction may have been limited given the sample size (N = 211) and the complexity of the moderated mediation model.

Fifth, the sample was restricted to four five-star hotels in a single Chinese tourism city, which limits the generalizability of the findings to other cultural contexts, hotel categories (e.g., mid-range or budget hotels), or geographic regions. Future research should replicate these findings in diverse settings.

Sixth, the qualitative phase employed convenience sampling, and although saturation was achieved, the findings may not represent the full diversity of experiences among tourism employees.

Seventh, despite efforts to reduce confirmation bias through independent coding, reflexive journals, and negative case analysis (see Section 4.1), the possibility of confirmation bias in the interviews and coding cannot be entirely excluded.

Eighth, measurement invariance across gender was not tested. Given that our analyses involved comparisons between male and female employees, future research should establish measurement invariance to ensure that the observed gender differences are not artifacts of differential measurement properties.

Ninth, the study focused on quantitative job insecurity (threat to job continuity). Future research should also examine qualitative job insecurity (threats to career opportunities, salary, and job autonomy) to determine whether different types of job insecurity have distinct associations with employee outcomes.

Tenth, employees were nested within four hotels. The small number of clusters (k = 4) precludes the reliable use of cluster-robust standard errors (Huang, 2026). Future research with a larger number of hotels should employ multilevel modeling to account for within-cluster correlation.

### 5.4. Conclusions

Driven by the trend of AI advancement and economic turndown, this study examined the conditions under which job insecurity is associated with customer orientation among tourism employees and provided plausible explanations for the observed patterns. The findings indicate that job insecurity was associated with lower customer orientation through two pathways: a direct association and an indirect association through reduced work engagement. The direct association was only significant among female employees with shorter industry tenure in this sample. The findings suggest that tourism employers should be attentive to employees’ feelings of job insecurity, particularly among junior female employees, and consider developing differentiated management strategies to address these concerns. Future research should further investigate the boundary conditions and mechanisms underlying the association between job insecurity and employee outcomes, ideally using multi-source data and longitudinal designs to strengthen causal inferences.

## Figures and Tables

**Table 1:** Results of confirmatory factor analysis.

Model	df	χ^2^	χ^2^/df	RMSEA	GFI	NFI	IFI	CFI	RMR
Three-factor model	140	262.91	1.88	0.06	0.90	0.95	0.98	0.98	0.06
Two-factor model a	142	950.71	6.70	0.17	0.74	0.81	0.84	0.83	0.25
Two-factor model b	142	940.74	6.62	0.16	0.75	0.81	0.84	0.84	0.24
Two-factor model c	142	458.48	3.23	0.10	0.82	0.91	0.94	0.94	0.07
One-factor model	143	1135.88	7.94	0.18	0.69	0.76	0.80	0.80	0.25

Note: Two-factor model a: (work engagement + job insecurity), customer orientation; two-factor model b: work engagement, (job insecurity + customer orientation); two-factor model c: job insecurity, (work engagement + customer orientation).

**Table 2:** Characteristics of the respondents.

Characteristics	N	%
Age		
17 and younger	4	1.9
18–30	103	48.8
31–50	97	46.0
51 and older	7	3.3
Gender		
Male	94	44.5
Female	117	55.5
Education		
Some School	1	0.5
Middle School	55	26.1
High School	15	7.1
College	136	64.5
Graduate degree	4	1.9
Position Type		
Non-managerial position	125	59.2
Managerial position	86	40.8
Industry Tenure		
Less than 6 months	20	9.5
6 months–1 year	29	13.7
1–2 years	28	13.3
2–3 years	23	10.9
3–4 years	9	4.3
4–5 years	11	5.2
More than 5 years	91	43.1

**Table 3:** Means, standard deviations, and correlation coefficients.

Variable	M	SD	1	2	3	4	5	6	7	8
1. Age	2.51	0.60								
2. Gender	0.45	0.50	−0.10							
3. Position Type	0.59	0.50	0.34 **	−0.01						
4. Education Level	3.41	0.91	−0.26 **	0.09	0.07					
5. Marital Status	1.75	0.62	0.24 **	0.10	0.22	−0.14 *				
6. Industry Tenure	6.07	3.85	0.60 **	−0.08	0.49 **	−0.05	0.17 *			
7. Job Insecurity	2.91	1.67	−0.05	−0.18 *	−0.20 **	−0.13	−0.04	−0.10		
8. Work Engagement	5.47	1.02	0.24 **	0.03	0.11	−0.09	−0.01	0.10	−0.28 **	
9. Customer Orientation	5.38	0.97	0.20 **	−0.03	0.15 *	−0.08	0.01	0.08	−0.31 **	0.73 **

Note: ** *p* < 0.05, * *p* < 0.10.

**Table 4:** Results of hypothesis testing.

Mediator Variable Model (Work Engagement)	B	SE	t	*p*
Constant	6.78 ***	0.61	11.15	<0.01
Age	0.19 **	0.07	2.68	0.01
Gender	−0.08	0.62	−1.19	0.23
Position Type	−0.03	0.16	−0.21	0.83
Education Level	−0.07	0.05	−1.34	0.18
Industry Tenure	−0.08	0.06	−1.39	0.17
Job Insecurity	−0.51 ***	0.13	−3.94	<0.01
Job Insecurity × Industry Tenure	0.03	0.02	1.85	0.07
Job Insecurity × Gender	0.37 **	0.18	2.05	0.04
Industry Tenure × Gender	0.03	0.08	0.38	0.71
Job Insecurity × Industry Tenure × Gender	−0.03	0.03	−1.28	0.20
R^2^ = 0.19 *** F−value = 4.21 R^2^ Change = 0.01				
**Dependent Variable Model (Customer Orientation)**				
Constant	3.99 ***	0.99	3.99	<0.01
Age	0.00	0.05	0.03	0.98
Gender	−2.09	1.28	−1.63	0.10
Position Type	−0.11	0.11	−1.05	0.30
Education Level	−0.01	0.04	−0.33	0.74
Industry Tenure	−0.07	0.13	−0.56	0.58
Job Insecurity	−0.44 ***	0.11	−4.04	<0.01
Work Engagement	0.51 ***	0.15	3.58	<0.01
Job Insecurity × Industry Tenure	0.04 ***	0.01	2.91	<0.01
Work Engagement × Industry Tenure	−0.01	0.02	−0.44	0.66
Job Insecurity × Gender	0.45 ***	0.14	3.25	<0.01
Work Engagement × Gender	0.16	0.20	0.82	0.41
Industry Tenure × Gender	−0.01	0.16	−0.04	0.97
Job Insecurity × Industry Tenure × Gender	−0.04 **	0.02	−20.40	0.02
Work Engagement × Industry Tenure × Gender	0.03	0.02	10.02	0.31
R^2^ = 0.61 *** F−value = 20.15				
R^2^ Change 1 (Job Insecurity × Industry Tenure × Gender) = 0.01 **				
R^2^ Change 2 (Work Engagement × Industry Tenure × Gender) = 0.002				

Note: *** *p*< 0.01, ** *p* < 0.05.

**Table 5:** Conditional direct and indirect effects of job insecurity on customer orientation.

		Direct Effect				Indirect Effect			
Industry Tenure	Gender	Coefficient	SE	LLCI	ULCI	Coefficients	SE	LLCI	ULCI
M-1SD (2.22 years)	Female	−0.35 ***	0.08	−0.52	−0.19	−0.18	0.15	−0.48	0.12
M-1SD (2.22 years)	Male	0.01	0.07	−0.13	0.13	−0.17	0.16	−0.49	0.15
M (6.07 years)	Female	−0.19 ***	0.05	−0.30	−0.09	−0.21	0.14	−0.49	0.07
M (6.07 years)	Male	−0.01	0.05	−0.11	0.09	−0.20	0.15	−0.50	0.10
M+1SD (9.92 years)	Female	−0.03	0.07	−0.17	0.10	−0.23	0.15	−0.53	0.07
M+1SD (9.92 years)	Male	−0.02	0.07	−0.16	0.11	−0.22	0.16	−0.54	0.10

Note: *** *p*< 0.01.

**Table 6:** Interview protocol.

Interview Question	Purpose and Rationale
How long have you been working in the hotel industry?	This question is used to confirm the validity that the interviewee is qualified for the study.
Do you like your job? How and why?	This question serves as an icebreaker to start the conversation.
How important do you think customer orientation is to your work? Why?	This question is a general question to start the conversation about customer orientation.
What factors would affect level of your customer orientation? Why?	This question is used to identify potential factors affecting the interviewee’s customer orientation.
Do you feel any job insecurity? Do you feel that your job opportunities or career development opportunities are under any threat?	To start the conversation about quantitative job insecurity (concerns about job loss and employment continuity).
What factors do you think contribute to your job insecurity?	To explore contributors to quantitative job insecurity (threats to job continuity).
Do you think your feelings of job insecurity would influence your level of customer orientation? Why?	To explore the perceived relationship between quantitative job insecurity and customer orientation.
What do you think hotel managers can do to improve their employees’ customer orientation? Why?	This question is used to explore additional findings on how to improve employees’ customer orientation.
What do you think hotel managers can do you mitigate their employees’ job insecurity? Why?	This question is used to explore additional findings on how to mitigate employees’ job insecurity.

**Table 7:** Profiles of the interviewees.

Participant	Gender	Age	Industry Tenure	Position
P1	F	22	1	Front-desk agent
P2	F	22	1	Telephone operator
P3	F	23	2	Bar server
P4	F	23	2	General manager assistant
P5	F	30	7	Front-desk supervisor
P6	F	49	15	Public area attendant
P7	M	21	1	Restaurant server
P8	M	23	2	Front-desk agent
P9	M	24	3	Bellman
P10	M	24	3	Restaurant server
P11	M	35	10	HR manager
P12	M	45	10	Housekeeper

**Table 8:** Thematic coding structure.

Theme	Sub-Themes	Definition	Representative Quotes
1. Job insecurity as a hindrance stressor	Fear of job loss; uncertainty about career future	Employees perceive job insecurity as a threat that depletes psychological resources and undermines work motivation	P1: “Whenever I think of my unsure future career, I have to wonder why I have to step forward to help my customers.” P2: “I believe feeling insecure on my job would affect my service quality.” P3: “After all, this job is not a ‘golden bowl’.”
2. Reduced engagement as a perceived process	Loss of focus; reduced absorption; diminished vigor	Job insecurity reduces employees’ ability to fully engage in and concentrate on their work	P2: “When you think of your unsure future career, you have to think about what other career opportunities you may have, and then you cannot focus on what you are doing.” P5: “I would not be able to be as absorbed in my work as usual when I worry about the work and promotion opportunities.”
3. Industry tenure as a buffer	Habitual customer orientation; career mobility; professional detachment	Longer industry experience provides resources (habits, skills, professional networks) that help employees withstand job insecurity	P5: “I have been working in this industry for 7 years, and trying to provide customer-oriented service has become part of my habits.” P6: “It is easy to find such a position in the market. If I lost this job, I can find one somewhere else.” P11: “Not letting personal feelings impair your work is a basic professional quality.” P12: “I am required to provide customer-oriented service. I have been used to do it.”
4. Gender differences in responses	Gendered structural constraints and situational demands shape different coping responses	Women and men may describe different coping responses because of unequal structural resources and caregiving demands.	P1: “It is not easy to get rid of this feeling of job insecurity which often distracts me from my work.” P7: “What I can only do to secure my job and career opportunities is to work harder rather than to worry.” P8: “I am just too busy to worry about job insecurity. I have to do multiple tasks every day and try to provide excellent service in every single task.”
5. Negative cases: No job insecurity	Labor shortage as security; AI limitations	Some employees do not perceive job insecurity due to external factors (labor shortage, limited AI capabilities)	P4: “In the tourism industry, employees are not the party who need to worry about security, but the employers are in the place of insecurity. I mean employers worry about unsecured labor force.” P6: “If I lost this job, I can find one somewhere else pretty soon.” P9: “I do not think my job can be replaced by machines in the near future… we only have robots deliver meals.” P2: “Robots make lots of mistakes. I do not think my job would be replaced by robots.”
6. The paradox of commitment	Career commitment increases vulnerability	Employees who are more committed to the tourism industry as a long-term career experience stronger job insecurity	P5: “I do not want my job to be replaced by technologies, after all I love my job… I often think about what I can do to be not replaced by robots.” P7: “I want to stay in this industry as my life-long career. Some of my tasks have been replaced by robots… I like rotation opportunities to learn more skills.”

## Data Availability

The data presented in this study are openly available at https://pan.baidu.com/s/1VvSCjhBloUJ7D7gdnIrFrA?pwd=9hr8 (accessed on 3 October 2026).
